# Profiles of Early Expressive Phonological Skills-Brazilian Portuguese (PEEPS-BP): a diagnostic accuracy study

**DOI:** 10.1590/2317-1782/e20240106en

**Published:** 2025-01-27

**Authors:** Simone Nicolini de Simoni, Denis Altieri de Oliveira Moraes, Karina Carlesso Pagliarin, Márcia Keske-Soares

**Affiliations:** 1 Programa de Pós-graduação em Distúrbios da Comunicação Humana, Departamento de Fonoaudiologia, Universidade Federal de Santa Maria – UFSM - Santa Maria (RS), Brasil.; 2 Programa de Pós-graduação em Distúrbios da Comunicação Humana, Departamento de Estatística, Universidade Federal de Santa Maria – UFSM - Santa Maria (RS), Brasil.

**Keywords:** Children, Speech, Language, Vocabulary, Validity

## Abstract

**Purpose:**

To present the criterion validity, sensitivity, specificity, and cut-off scores for the Profiles of Early Expressive Phonological Skills Test - Brazilian Portuguese (PEEPS-BP) - Expanded List.

**Methods:**

This was a quantitative cross-sectional psychometric study. The sample consisted of 30 children with no identified neurodevelopmental disorders aged 24 to 36 months. Twenty-three were part of the control group, and seven were part of the clinical group, which consisted of children with reported delays in vocabulary acquisition and phonological development. Children were administered the PEEPS-BP - Expanded List and responses to each item/stimulus were analyzed based on the following categories: spontaneous naming, repetition, and not naming the item at all. Criterion validity was established using Student’s T-test to compare the scores obtained by clinical and control group participants on the instrument. Sensitivity and specificity analyses were performed using Receiver Operating Characteristic (ROC) Curves. Results were considered significant at p ≤ 0.05.

**Results:**

Scores of the clinical and control groups differed significantly at p ≤ 0.001. A cut-off point of 34 had a sensitivity of 0.957 and specificity of 100.00 in distinguishing between the two participant groups.

**Conclusion:**

The PEEPS-BP had adequate criterion validity and cut-off points that could distinguish between the clinical and control group with satisfactory sensitivity and specificity.

## INTRODUCTION

Vocabulary acquisition and the emergence of first words mark the point at which children can begin to engage in social interaction through oral communication. This period is associated with specific developmental landmarks^(1)^. The first words are acquired at approximately 12 months of age, after which word acquisition continues at a slow and gradual pace of 10 additional words per month until the age of 18 months. When the child’s vocabulary expands to a total of 50 words, near 24 months of age, the speed of word acquisition increases, and a progressively larger number of words can be used in oral communication^(2)^.

In Brazilian Portuguese (BP), the nasals (/m, n, ɲ/) are acquired first, followed by the plosives (/p, b, t, d, k, g/) and some fricatives (/f, v, s, z/), all of which are mastered before three years of age. The phonemes /ʃ, ʒ/ are acquired at three years six months and mastered at four years of age. The liquids /l/ and /X/ are also mastered at three years and six months. Particularly in this reference, the phoneme /ʎ/ is acquired at seven years of age and mastered at 8:6, . The phoneme /ɾ/ is acquired at four years of age and at the age of 4:6 are mastered^(3-5)^. However, there are other important studies in Brazilian Portuguese literature that present similar data on phonological acquisition, differing in the method adopted in each study.

The assessment of phonology and vocabulary in small children presents a challenge for researchers and clinicians, as few instruments are available in the national or international literature to evaluate linguistic development and diagnose the presence of impairments, especially in young children below the age of 3 years^(6)^.

In Brazil, no instruments are available to assess vocabulary and phonology together in the same instrument, in children younger than three years of age. In English, however, the authors Stoel-Gammon and Williams^(7)^ created the Profiles of Early Expressive Phonological Skills (PEEPS) test, which evaluates phonology and vocabulary in children aged 18 to 36 months. This test contains two lists of stimuli: the basic list, for children aged 18-36 months; and the expanded list, for children aged 24 to 36 months. The lists contain a different number of words to reflect the age-related growth in lexical knowledge. The test is administered using three-dimensional toys to represent the stimulus words, so it is in a playful version.

The PEEPS-BP Basic Word List was adapted by Oliveira^(8)^ in a study of typically developing children and children with cleft lip and/or palate aged 18 to 36 months. The PEEPS-BP Expanded Word List has also been adapted and has demonstrated satisfactory content validity^(9)^. The content validity study yielded a final version of the expanded list containing 29 words from different semantic categories that are part of children’s vocabulary. The words also contain BP phonemes in different word and syllable positions (Initial Onset, Medial Onset, Complex Onset, Medial Coda, Final Coda). The PEEPS-PB - Expanded List contains 29 words: *balão* (balloon), *banana, barriga* (belly), *boca* (mouth), *boneca* (doll), *cabelo* (hair), *cachorro* (dog), *cama* (bed), *chapéu* (hat), *colher* (spoon), *copo* (glass), *dente* (tooth), *elefante* (elephant), *fralda* (diaper), *leão* (lion), *língua* (tongue), *mamadeira* (baby bottle), *mão* (hand), *orelha* (ear), *pé* (foot), *peixe* (fish), *sabonete* (soap), *sol* (sun), *suco* (juice), *caminhão* (truck), *lua* (moon), *meia* (sock), *pente* (comb) and *perna* (leg). This list should be administered to children aged 24 to 36 months together with the Basic Word List to accommodate the phonological and vocabulary skill levels of children in this age group.

All this information about the content validity of the PEEPS-BP expanded word list, was published scientifically, presenting the composition and selection of target and representative words for the instrument. Also, in the article there are other psychometric criteria adopted in this stage of content validity^(9)^.

Psychometric studies of the PEEPS-BP - Expanded list are required for the instrument to be used in clinical practice and research. Such studies should evaluate its criterion validity, sensitivity, specificity, and cutoff points to provide evidence of the test’s ability to distinguish between typical and atypical development of the constructs under study, which in this case, consist of receptive and expressive vocabulary.

This study aimed to examine the criterion validity (sensitivity and specificity) and cutoff point of the receptive and expressive vocabulary scores on the PEEPS-PB - Expanded List. The phonological results of the instrument will not be presented in this study, as they present a different analysis in relation to specificity and sensitivity, since the data bases needs to go through the analysis of judges, quantitative and qualitative calculations and will be developed in another scientific study. Furthermore, there is an answer sheet validation process similar to the original test in which phonological data is added. This does not invalid the presentation of the diagnostic accuracy study.

## METHODS

### Study design

This was an exploratory, quantitative cross-sectional study that used psychometric methods to establish the criterion validity of the PEEPS-BP - Expanded List.

This study was performed as part of a research project approved by a university research ethics committee, by the number 18419313.30000.5346. Written informed consent was obtained from the guardians of all participating children before the start of the study, as recommended by National Health Council Resolution 466/12. Additionally, as recommended by the International Test Commission guidelines (ITC- 2017), the permission of the authors of the original PEEPS was sought before the instrument was adapted to BP.

### Participants

The sample contained 30 children of both sexes, aged 24 to 36 months, recruited by convenience since the study occurred during the COVID-19 pandemic.

The control group (GCon) contained 23 children. The parents responded to a general medical history interview and did not report any issues in their children’s development. Additionally, they completed the MacArthur inventory^(10)^ and indicated that their children had produced at least ten of the words listed in the instrument. Furthermore, the children were expected to present otoacoustic emissions and to be rated as “competent” on all subscales of the Bayley Scale^(11)^- Screening, which evaluate cognition and motor skills in addition to receptive and expressive language.

The clinical group (GClin) contained seven children. In the medical history interview, the parents of these children reported them to have delayed speech acquisition, either in terms of speech production or vocabulary, but otherwise neurotypical functioning. Additionally, though they were able to produce few of the words listed on the MacArthur Inventory^(10)^, this figure was still equal to or greater than ten words. The children also had otoacoustic emissions. Children in the GClin were rated “competent” on the cognitive, motor, and receptive language subscales of the Bayley Scale^(11)^ - Screening, but their expressive language skills were classified as “delayed” or “emergent”. Table 1 shows the sex and age distribution of the sample.

**Table 1:** Sex and age distribution of the sample

	Clinical Group	Control Group
	(n = 7)	(n = 23)
Gender F/M	2/5	13/10
24-30 months	6	7
31-36 months	1	16

Caption: F = female; M = male

### Procedures

The medical history interview was performed online through Google Meets. The interview contained questions on pre, peri and postnatal development; landmarks of motor and oral language development (including early vocalizations, babbling, first words); general health; and environmental factors.

Parents/guardians also completed the BP version of the MacArthur Inventory^(10)^, which assesses expressive vocabulary in different semantic classes in children aged 16 to 36 months. When completing this instrument, parents were asked to identify which words were present in their children’s expressive vocabulary.

Auditory assessments were performed in person using the Transient Evoked Otoacoustic Emissions (TEOAEs) test. This procedure was performed using the Otoread system to assess transient emissions and adequate cochlear function, confirming that the child could hear. The response criterion established by the device is an outcome of “pass,” for both ears.

The Bayley Scale^(11)^ - Screening, considered the gold standard for developmental assessment, was administered in-person to provide an age-based rating of children’s cognitive development, gross and fine motor skills, as well as receptive and expressive language. The results of each subscale were classified as “competent”, “delayed” or “emergent” using age-based parameters.

All assessments were carried out by the main author of this study, both the hearing test and the application of the Bayley Scale. To apply the Scale, the author of the research has the necessary certification, according to the Scale’s requirements, as well as the necessary material certified by the company test. For the Bayley Scale^(11)^, all assessments were films and scores were performed by the main author and reviewed by another professional also certified with the scale.

After performing these assessments and determining whether children would enter the study as participants of the GClin or GCon, the PEEPS-BP-Expanded List was administered in person. The stimulus words from the Basic List^(12)^ were used together with those from the Expanded List to examine the criterion validity, sensitivity, and specificity of the latter. The PEEPS-BP-Expanded list was administered in a structured, air-conditioned room, ensuring the comfort of the child. The room contained floor mats, a Sony video camera, and a Panasonic audio recorder. The PEEPS-BP-Expanded list is administered using toys that correspond to words in the basic and expanded lists^(12)^. The toys were placed in boxes and grouped by semantic category or contextual association. The boxes were laid out in a predetermined order, but the child was allowed to select them at random. Spontaneous naming was encouraged each time an object was selected. If the child did not spontaneously name the objects, the examiner would elicit the word through repetition. The test was administered by the examiner, who offered the stimulus objects to the child in a playful context, providing instructions and phrases to encourage the production of stimulus words, as needed.

During the assessment, the child was invited into the data collection room for the PEEPS-PB-Expanded List with the examiner and their guardian, and the following instruction was given:

*I will show you the toys that are in these boxes, and you should tell me the name of each toy if you know them. When we finish looking at all the boxes with the toys, you can play with all of them with your mother/father/guardian*.

The child would then be allowed to randomly select the boxes, while the examiner used sentences to encourage spontaneous naming if necessary, as suggested in the original test manual. The child would go through all boxes and name each item - spontaneously or through repetition - until the PEEPS-BP-Expanded List was completed.

The videos were analyzed and children’s responses during the test were rated. Each of the 29 words in the test was given one of the following scores: spontaneous naming - two points; imitation or repetition - one point; not naming the item - zero points. This calculation yields a maximum possible score of 58.

### Data analysis

Student’s T-test was used to analyze the difference between control and clinical groups. The *Receiver Operating Characteristic* (ROC) *curve* was used to determine the sensitivity and specificity of various cut-off points for the PEEPS-BP. Data were analyzed using SPSS^(13)^ and results were considered significant at p ≤ 0.05.

## RESULTS

Scores on the PEEPS-BP - Expanded List differed significantly between the GCon and GClin (Table 2). Children with delayed vocabulary and expressive language development achieved lower item-naming scores than those with typically developing expressive vocabulary.

**Table 2:** Comparison of scores obtained by the GCon and GClin on the PEEPS-BP - Expanded List

PEEPS-BP - Expanded List	Group	n	Mean	Standard deviation	t	p
Correct responses	Control	23	50.957	8.177	8.236	≤0.001
	Clinical	7	23.429	5.804		

Caption: t = T-test for two independent samples; p = Exact probability of a Type I error assuming the null hypothesis is true

The analysis of ROC curves revealed an area under the curve of 0.994 which indicates a high degree of accuracy. In other words, the Expanded List can effectively distinguish between the control group and the clinical participants, who have impairments in oral language. The sensitivity and specificity values for different cut-off points are shown in Table 3. The 34-point cut-off had a sensitivity of 95.7% and specificity of 100%.

**Table 3:** Sensitivity and specificity of the total score of the PEEPS-BP - Expanded List

Cut-off point	Sensitivity (%)	Specificity (%)
17.5	100.0	14.3
22.0	100.0	42.9
24.0	100.0	57.1
26.0	100.0	71.4
28.0	100.0	85.7
31.0	95.7	85.7
34.5	95.7	100.0
37.5	91.3	100.0
40.0	87.0	100.0
41.5	82.6	100.0
43.5	78.3	100.0
47.0	73.9	100.0
51.5	69.6	100.0
54.5	52.2	100.0
55.5	26.1	100.0
56.5	17.4	100.0
57.5	13.0	100.0
59.0	43.0	100.0

## DISCUSSION

The present findings regarding the criterion validity, sensitivity, specificity, and cut-off points of the PEEPS-BP - Expanded List contribute to the instrument’s cross-cultural adaptation process as they demonstrate its ability to distinguish between the expressive vocabulary scores of the GClin and the GCon. These results support the criterion validity of the PEEPS-BP - Expanded List and the sensitivity and specificity with which it can distinguish between typical and atypical children, that is, those with delays in expressive vocabulary acquisition.

Few studies in the Brazilian literature have conducted simultaneous phonological and vocabulary assessments using quantitative scores that can differentiate between typically and atypically developing individuals^(14,15)^. According to the vocabulary context, atypically-developing children identified at an early age showed a reduced vocabulary which did not correspond to their expected level of oral language development. This is indicative of expressive language difficulties, as their performance was worse than that of typically-developing children, who were able to name a higher number of test items.

Early vocabulary assessments allow speech pathologists to observe whether a child’s performance is within expectations for their age^(16)^. In the PEEPS-BP - Expanded List, this corresponds to an accuracy rate of at least 50% or 34 points. Hage and Pereira^(17)^ note that at 24 to 36 months, children with fewer than 50 words in their vocabulary can be classified as having difficulties or delayed vocabulary acquisition.

Atypically developing children, who do not achieve the expected cut-off scores, cannot access the receptive vocabulary required to perform the spontaneous naming task, and therefore make omission errors and phonological substitutions^(18,19)^. In other words, to be classified as typically developing, a child’s vocabulary must be large enough to encompass the stimulus words^(20,21)^, as this suggests an adequate level of expressive vocabulary and speech sound production.

The culturally adapted Brazilian version of the PEEPS-BP - Expanded List may be an adequate instrument to evaluate linguistic aspects of child development, especially vocabulary acquisition, in children aged 24 to 36 months. In this investigation, the instrument’s criterion validity, sensitivity, and specificity were evaluated to determine its ability to distinguish between the performance of a clinical and a control group. This process helped us achieve the goal of demonstrating the applicability and efficacy of the PEEPS-BP - Expanded List.

The PEEPS-BP test was adapted to assess both the phonological and lexical aspects. The adaptation process followed the original test creation process, taking into account the analysis of the types of responses. The types of responses for the overall test score were divided into a scale of responses of 0 points, 1 point and 2 points. This scale makes us reflect on the following response configurations: 0 points, the child does not actually have the target word represented by the toy in his/her lexical repertoire or, as a limitation of the test, did not recognize the toy, but through the repetition analysis that generates 1 point, is able to identify or recognize it.

The type of response that configures 1 point cannot be discarded or invalidated, since it demonstrates that the child is able to repeat the vocabulary, demonstrating recovery of lexical access to the repertoire, performs phonemic production and the child still experiences the stimulus of the target word^(18)^. It should be noted that, the entire discussion that permeates the response scale, takes place in the midst of the playful context and the child makes this naming while playing and the offering of toys, as exemplified in the study method, is carried out using toys with similar semantic categories.

Also, the PEEPS-BP test with vocabulary analysis, does not measure the semantic context in its literal sense, that is, it does not assess whether the child knows what a given target word is for or how it works based on the representation of the toy, but rather analyzes lexical knowledge and access. This also applies to the standard response of 1 point for repetition, which is different, for example, if the evaluator asked the child where a given object is and the child simply pointed. In this sense, the vocabulary assessment in other Brazilian Portuguese tests, along the same lines, also considers the repetition of the item presented, but according to the analysis of each test.

This shows that the child can, through the process of repeating a stimulus, possibly acquire or increase his/her vocabulary. Study shows that the more a child is exposed to different stimuli and words, the more predisposed he/she will be to incorporate them into his/her expressive vocabulary^(22)^. The fact that repetition occurs in the PEEPS-BP response scale leads us to reflect on the cognitive predisposition and recovery of lexical access that the child presents to speak at the time of the assessment, therefore, the type of response must be considered.

No less important is the discussion of this work, that should permeate the age range presented between 24 and 36 months, a period in which, according to the main studies in the literature, the child is in full vocabulary development, called the ‘’vocabulary explosion”; for this reason, the types of responses of the child to the test must be taken into consideration, thinking about this full lexical improvement^(1,2)^. Access to linguistic input and the process of quickly recognizing the word is associated with simultaneous knowledge of vocabulary, a fact that also goes against the analysis of the original test that has its scale of response types in: spontaneous response, repetition/direct imitation and non-response to the item^(7)^.

It is known that every study and psychometric process involving stages of adaptation of an instrument present weakness. Therefore, applications in different contexts and situations elucidate a work in search of its reliability. The PEEPS proposal of using toys to represent the target words becomes a different strategy because it allows the child to enjoy the playful context for the moment of evaluation. However, the representativeness of the object may at some point compromise the veracity of the response, since different objects/toys can represent the same semantic item.

In this sense, studies involving children with very young ages are more difficult to conduct, since they impact an effective cognitive and linguistic assessment process and an analysis of responses that considers the period of oral language acquisition, without discarding the focus on the risks and delays in the process of child linguistic development. Therefore, it is expected that PEEPS-BP will assist and contribute to other scientific and academic research and also to reasoning and assessment in speech therapy clinics.

However, it is important to collect additional evidence of this instrument’s psychometric property in larger samples, to encourage future research on the phonological and lexical aspects of oral language development.

## CONCLUSION

The PEEPS-BP-Expanded List had adequate criterion validity and was able to distinguish between the expressive vocabulary scores of typical and atypical children. Furthermore, it had sufficient sensitivity and specificity to identify children with receptive and expressive vocabulary alterations.

In this sense, the psychometric questions were designed to verify the criterion validity, as well as data regarding the development of aspects of oral language, such as expressive vocabulary, were able to be evaluated and analyzed based on the application of the test and verification of the results.

However, whenever cross-cultural adaptations or even the creation of new instruments are made, regional and sociodemographic differences and access to different economic classes in the country need to be considered. In this sense, for the population evaluated with this test, we present these results, not being applied in other regions of the country, characterizing it as a limitation of the study.
